# The Doctor in the West-End Mews

**Published:** 1888-09-22

**Authors:** E. Lloyd Jones


### The Doctor in the West-end Mews.

By E. Lloyd Jones, M.B., B.A., Cantab.

Some of the poorest inhabitants of the whole metropolis live in the West-end: like a giant parasitic undergrowth, they congregate about the luxurious homes of the wealthy, upon whose employment they depend, or by whose charity they are just kept from starvation.

The aim of the present paper is, to give some account of the experiences of a medical man whose time during some months was devoted to sick visiting among the poor in this district.

The West-end poor affect mainly three kinds of habitation —the common lodging-houses, the so-called "model dwellings," and the mews. The first may be described as unsavoury and unclean; the second are more unsavoury and more unclean; the third are most unsavoury, and they would, I doubt not, be more unclean than their rivals were such a state of things in any degree possible.

The common lodging-houses are lofty, and present many stairs to the upward aspirant; the model dwellings attain still higher altitudes; while the stable habitations in the mews recommend themselves by the fact that they never attain to a third storey, for their strength would not uphold the additional burden. During my tenure of office I paid about two thousand visits in three months, which means, by a rough calculation, an ascent of about six hundred feet per day, or about thirty-nine miles achieved per annum in the celestial direction. I shall refer no more to the common lodging-houses or the model dwellings; it is of the mews and their sick inhabitants that I shall more especially speak in the rest of this paper.

A mews appears to have been originally built for the accommodation of horses and poultry, and for the storage of vehicles, and seems to serve but secondarily for the habitation of necessitous and dependent man. After entering one, and picking your way between children, poultry, dogs, and pools of stale filthy water, you find the house to which you are summoned. Walking into the stable, you inquire for your patient, and are directed to a flight of wooden steps, by which you are to ascend to the stable-loft above. One might describe an endless variety of these approaches—step-ladders of which most of the steps are missing, treacherous enough in the dark, step-ladders inside the stable, step-ladders outside the stable, ladders which deposit you at the foot of a tenanted dog-kennel, ladders which have their embouchure in a pig-stye.

As you are about to enter the room, which is pervaded often by a characteristic ammoniacal odour, a murmur "The doctor" is heard from within, warning you that the inmates are preparing for your arrival. A minute ago a family were sitting round the table; now they are scattered about the room—some wiping their mouths with their fingers, and all trying to appear unconscious of their surroundings, and at ease, as if they were not actually ashamed to have been caught eating. The table a minute ago supported a plate of red herrings, some turnip-tops—the last a very popular dish—and a large jug of beer; now, a cloth hastily spread about it only discloses the shape of a beer-jug and the outline of a pie-dish, which have been hastily hidden from view. Glancing round the room, you observe that it is ill-lighted, there being only one small window at each end: the walls are whitewashed, the furniture common and bare, the floor uncovered, and through its crevices you hear the sounds of the munching quadrupeds below. On the walls are a few cheap chromos, and you are puzzled to decide which cost the least, the pictures or their gorgeously gilded frames. The room is, as a rule, dirty—sometimes, however, not very dirty. There is seldom any place where you like to deposit your hat, for the table is generally strewed with slops, and it is inexpedient to rest a hat where it may become tenanted by the too bold "hemiptera." There is often a collection of modelled fruit under a glass shade, fruit having the clear complexion which in genuine fruit cometh only of rottenness. You make a bee-line to the heart of the family if you exclaim with sufficiently well-feigned admiration, "That *is* fruit!"

On turning to the patient and asking, "What is the matter?" you will frequently be told that they do not know; that they sent for you so that you might tell them what was the matter. My next question has often been, "What does she complain of?" And the reply has often been a laconic "Nothing." After a little preliminary fence of this kind one generally extracts a great many crooked answers and sufficient information to afford a clue to the nature of the illness. These patients are as a rule polite, so far as knowledge lends them power, and studied kindness and politeness never fail to win their hearts. They are not used to such treatment, and it has all the potency of a new weapon.

The clergy, and almost as frequently the doctor, are regarded often as vehicles for maternal ministration, and the most popular Church is the one which affords most physical consolation. Many belong to the Roman Church, and on Sundays they are to be found reading "The Garden of the Soul," or admiring pretty texts in French or Latin, which they cannot translate, but which they will often ask you to translate for them. An old drunkard said to me one day, "Doctor, they've been asking me to see the parson. I said I don't want to see the parson. I always say let's first see what the doctor can do."

Many are sufficiently sober, honest, and thrifty, but a large percentage are addicted to drink—dishonest, thriftless, and idle, and nearly all have a most egregrious lack of commonsense, due, I take it, to deficient education: they have never been taught to think. They will give the most abominable articles of food to sick persons. The mother of a young girl who had scarlet fever called me up at one a.m. on one occasion to ask if her daughter might have some "mackerel soused in vinegar."

If you tell them to stay in bed, and you visit again at an unexpected hour, you will find your patient up and about, and may hear that he has been seen in the public-house round the corner. If he is in, he will tell you that he "only got up while the bed was being made," and if you surprise him again two hours after, he will be up again, and fluent with excuse.

There is an amusing side also to one's experiences among these poor Londoners. Many suffer from fancied ailments. One of these pseudo-sufferers, at the end of a tedious recital of aches and pains, burst into tears, and said, "Doctor, nobody knows what I *seem* to suffer."

Another robust-looking individual showed me the outlines of a well-developed muscle, and asked, "Doctor, is this the cancer?"

One day I found a sweep standing up in a small foot-bath containing about a gallon of water. I asked "Do all sweeps wash every night before they go to bed?" He drew himself up and replied, proudly, "No, sir; they have'nt all got the accommodation."

Sometimes these patients write to you, and their letters are generally amusing and often very difficult to decipher. Here are two examples:—

"Sir,—Would you kindly give me some more strengthen medicine the after good medicine and more of sena and it was not mixed with the peper last time please will you have it mixed.—Your servant, Betsy Grumble.

The *peper* referred to the peppermint she was so partial to. Here is a piquant piece of composition :—

" Dear Doctor,—I shall be glad if you will come to see me as soon as possable. I don't think my case or my wifes neglecting in attendance or medicine will suit yours truly
"P. Q."

I have spoken of the homes and habits of the West-end poor. Let us glance at them in their parasitic aspect, depending, as they do, upon the employment and support by, or charity of, their wealthier neighbours. Although not a few are employed as coachmen, chimney-sweepers, or fulfil other functions, there are a very large number—an astonishing number—who are almost always out of work or invalided, and these subsist upon charity.

A large percentage of these have especial "patrons," by whom they are frequently spoiled and pampered until they lose any undeveloped germ of self-reliance they may have had, and perceive that it is worth their while to continue in as bad health as appearances permit. One of my patients was taken drives in the park almost every day, calling at her patron's house, where she reclined on a sofa and ate jelly before being driven again to her back attic.

When first a patient told me that "Lady Browne" was coming, I felt a secret and perhaps ill-concealed desire to retreat; but, after missing several such encounters, I was one day surprised, and found that "Lady Browne" was the green-grocer's wife from round the corner. The lady patron is generally dignified with the appellation of Lady So-and-So.

The patron is, however, often a lady in the accepted sense of the term—one who has a superfluity of money and time; and who visits from disinterested and generous impulses. By their refining influences alone these ladies must do a vast amount of good ; the harm they do is, perhaps, inseparable from any charitable system. They are frequently a source of much trouble to the busy doctor, writing long letters full of queries about poor Mrs. So-and-So, or dear Mrs. That; and they little think what a burden it is to the medical man to answer them, or they would call to see him, and save him the trouble of writing.

They often ask if "the doctor thinks that Mrs. Breakback had better take electric baths ?" or "if Cockle's pills would do any good to Mrs. Cruikshank?" adding that a sister of their coachman's "suffered from very similar symptoms," and was relieved by so-and-so. They do not mean to be impertinent. The most common query is such as this : "Would dear Mrs. Nebriate benefit by taking port wine ?"

I had a patient who provided his friends with port and whiskey, and who held quite cheerful alcoholic "at homes," and always *offered* a glass of something to his weary medical man. This man got but little to eat, but he was kept in spirits by some neighbouring relief committees, a rector—or two I should think—and a few lady visitors.

One of my patients, a very old woman and a dear old soul, had a visitor who paid her a call once a day without omitting even bank-holidays. She brought with her a few times a year four shillings from a benevolent society ; but as she "took tea" with the poor woman every day, the expenditure for tea and bread more than counterbalanced the amount of the donation, and her voracity, together with the trouble entailed in getting the meal ready, made my patient very anxious to see the last of her indefatigable visitor. Many a time have I found this old woman lamenting the recent depredations which had made such a substantial hole in the loaf of bread. This patient by the way paid for her lodging, for the services of a servant, and for food out of ten shillings a week, and she subsisted largely upon bread and chutney. She had the good sense to avoid medicine unless she was recommended to take it by her medical man.

The patrons sometimes lend books to their protegés. These are generally the rising generation of lady-visitors. I found a hysterical girl reading one entitled "The Broken Wedding-ring." I never yet saw one good book so lent, though I have found a few poor patients with libraries of several hundred standard works.

In concluding, I must confess to having taken a one-sided view of the habitations, manners and customs of the sick poor. If I have appeared to some to have criticised too freely the methods of the charitable, it has been from a desire to point out where charity often faileth, rather than from any want of sympathy with the objects of those who, in all kindliness and generosity, endeavour to send some rays of comfort into the homes and lives of our London poor.

